# RNA Splicing Factors and RNA-Directed DNA Methylation

**DOI:** 10.3390/biology3020243

**Published:** 2014-03-26

**Authors:** Chao-Feng Huang, Jian-Kang Zhu

**Affiliations:** 1 State Key Laboratory of Crop Genetics and Germplasm Enhancement, College of Resources and Environmental Sciences, Nanjing Agricultural University, Nanjing 210095, China; 2 Shanghai Center for Plant Stress Biology, Shanghai Institutes for Biological Sciences, Chinese Academy of Sciences, Shanghai 200032, China; 3 Department of Horticulture and Landscape Architecture, Purdue University, West Lafayette, IN 47907, USA; E-Mail: jkzhu@purdue.edu

**Keywords:** splicing, DNA methylation, heterochromatin formation, siRNA, epigenetic regulation

## Abstract

RNA-directed histone and/or DNA modification is a conserved mechanism for the establishment of epigenetic marks from yeasts and plants to mammals. The heterochromation formation in yeast is mediated by RNAi-directed silencing mechanism, while the establishment of DNA methylation in plants is through the RNA-directed DNA methylation (RdDM) pathway. Recently, splicing factors are reported to be involved in both RNAi-directed heterochromatin formation in yeast and the RdDM pathway in plants. In yeast, splicing factors may provide a platform for facilitating the siRNA generation through an interaction with RDRC and thereby affect the heterochromatin formation, whereas in plants, various splicing factors seem to act at different steps in the RdDM pathway.

## 1. Introduction

Cytosine DNA methylation is widespread in eukaryotes and plays critical roles in diverse biological processes including development [1,2,3,4], the silencing of transposons and other DNA repeats [5,6,7], X-chromosome inactivation in mammals [8,9], and genomic imprinting [10,11,12,13]. In mammals, nearly 25% of all methylated cytosines occurs in non-CG contexts (mCHG and mCHH, where H = A, C or T) in embryonic stem cells, more than 99% of methylcytosines are in CG context in differentiated cells such as fetal fibroblasts and approximately 70%–80% of CG dinucleotides are methylated throughout the genome [14,15]. By contrast, in plants, cytosine methylation in non-CG contexts can reach an appreciated level, with 23% in CHG and 22% in CHH context in immature floral tissue [16]. In adult leaves, the model plant *Arabidopsis thaliana* also has high levels of DNA methylation in non‑CG contexts, with 24% of CG, 6.7% of CHG and 1.7% of CHH methylation in the genome [17]. Unlike mammals in which DNA methylation is present throughout the genome [15], plants contain DNA methylation predominantly at transposons, other repeat sequences and centromeric regions [18].

In mammals, DNA methylation is catalyzed by DNA methyltransferases (DNMTs). DNMT1 is responsible for maintaining the symmetric CG methylation, and DNMT3A and DNMT3B are responsible for *de novo* DNA methylation [2,19,20,21]. In plants, maintenance of symmetric CG methylation is catalyzed by the DNA METHYLTRANSFERASE 1 (MET1) enzyme, an ortholog of DNMT1 [22]; the symmetric CHG methylation is maintained by a plant-specific DNA methyltransferase, CHROMOMETHYLASE 3 (CMT3) [23,24]; the asymmetric CHH methylation is maintained by DOMAINS REARRANGED METHYLTRANSFERASE 2 (DRM2), a homolog of DNMT3A/DNMT3B [23,25]. *De novo* DNA methylation in plants is guided by small interfering RNAs (siRNAs) in a pathway known as RNA-directed DNA methylation (RdDM) and DRM2 is the enzyme required for *de novo* methylation and catalyzes cytosine methylation in all three sequence contexts [23,25,26,27].

In the RdDM pathway, two plant-specific RNA polymerases, Pol IV and Pol V, are involved. Pol IV and Pol V act at different steps of this pathway, with Pol IV being required for 24-nucleotide (nt) siRNA biogenesis and Pol V functioning as a downstream effector for DNA methylation [28]. With the assistance of the SNF2-like putative chromatin remodeling protein CLSY1 and the homeodomain transcription factor-like DTF1/ SHH1, which interacts with Pol IV, Pol IV is recruited to transcribe transposons and repeat loci [29,30,31,32,33]. The resulting transcripts are copied into double-stranded RNAs (dsRNAs) by RNA-DEPENDENT RNA POLYMERASE2 (RDR2) and then processed into 24-nt siRNA duplexes by DICER-LIKE 3 (DCL3) [34,35]. Subsequently, the RNA methylase HEN1 methylates the siRNAs at their 3'ends for stability and then one strand of the siRNAs is loaded into AGO4 [36,37,38]. Pol V produces the nascent transcript to recruit siRNA-bound AGO4, through base‑pairing between the siRNA and nascent transcript [39]. The stable association of AGO4 with the Pol V transcripts is also dependent on its interactions with the largest subunit NRPE1 of Pol V and KTF1, a homolog of yeast transcription elongation factor Spt5 [40,41,42]. A putative chromatin-remodeling complex termed DDR, which is consisted of DRD1, DMS3 and RDM1 proteins, is required for Pol V association with chromatin and Pol V transcription [28,43,44]. The association of RDM1 protein of DDR complex with AGO4 and DRM2 may help to recruit DRM2 to Pol V-target regions for catalyzing DNA methylation [28,43,45].

Pre-mRNA splicing is an essential process required for the expression of most eukaryotic genes. Splicing is carried out by a macromolecular machinery termed the spliceosome, which senses the splicing signals and catalyzes the removal of introns from pre-mRNAs. The spliceosome is comprised of four small ribonucleoprotein particles (snRNPs), U1, U2, U4/U6 and U5 snRNPs, and numerous snRNP-associated proteins [46,47]. The spliceosome assembles on each intron through an ordered and highly co-ordinated pathway. The U1 snRNP and the heterodimeric splicing factor U2AF (U2 snRNP auxiliary factor) recognize the 5' and 3'-splice sites, respectively, to initiate the pre-spliceosome assembly, and then the U2 snRNP is recruited to the branch point of the introns through an interaction with U2AF to form the pre-spliceosome. Subsequently, the mature spliceosome is formed by the recruitment of U4/U6.U5 tri-snRNP to the pre-spliceosome and then the complex rearranges to form the catalytically active conformation after the release of U1 and U4 snRNPs [47,48]. Besides the core snRNPs, the spliceosome complex also contains a myriad of non-snRNP-associated splicing factors, with an estimation of upwards of 300 proteins [47]. It remains a challenge to characterize the function of the numerous non-snRNP-associated splicing factors.

In addition to the canonical function of splicing factors in pre-mRNA splicing, splicing factors might also play important roles in other biological processes. In support of this view, a group of splicing factors were reported to be involved in the RNAi-directed silencing process in fission yeast [49,50]. In parallel with the RNA-induced heterochromatin formation in *S. pombe*, recently, several splicing factors were shown to be involved in the RdDM pathway in plants. In this review, we will discuss the roles of splicing factors in RNA-directed silencing in yeast and RdDM pathway in plants, with emphasis on the distinct roles of different splicing factors in the RdDM pathway.

## 2. RNA Splicing Factors and RNAi-Directed Heterochromatin Formation in *S. pombe*

In *S. pombe*, heterochromatin is largely concentrated at *dh* and *dg* repeats and transposons that surround the centromeres, and the RNAi machinery is involved in the assembly of heterochromatin [51,52]. Biochemical studies in *S. pombe* have provided direct links between RNAi proteins and heterochromation [53]. The chromodomain protein Chp1, which is required for heterochromatic silencing, is shown to be associated with Argonaute (Ago1) in the RNA-induced transcriptional gene‑silencing (RITS) complex that also contains the GW-motif-containing protein Tas3 and siRNAs derived from *dg* and *dh* transcripts [53]. Through base-pairing of the siRNAs with nascent repeat transcripts transcribed by Pol II, the RITS recruits histone-modifying enzymes such as the H3K9 methyltransferase Clr4 to induce H3K9 methylation and the formation of heterochromatin [54,55,56]. The heterochromatin assembly in *S. pombe* involves a self-enforcing loop wherein siRNAs generated from heterochromatin feedback to facilitate the recruitment of heterochromatin-modification complexes [54,57]. In this mechanism, Pol II transcribes the *dg* and *dh* repeats and then Rdp1-containing RNA‑dependent RNA polymerase complex (RDRC) copies the transcripts into double stranded RNAs, which are processed by Dicer (Dcr1) into siRNAs [51,52]. The siRNAs are loaded into RITS complex, and the RITS localizes to heterochromatin region through the binding of Chp1 chromodomain to H3K9me and facilitates the association and recruitment of RDRC to nascent repeat transcripts transcribed from *dg* and *dh* repeats [57,58,59].

Through a screen for mutants with attenuated silencing of marker genes inserted in the *dg* region of *S. pombe*, Bayne *et al.* cloned a dozen of genes involved in the silencing of marker genes, which includes two genes, *Cwf10* and *Prp39*, that encode splicing factors [49]. To further examine the possible links between RNA splicing and centromere silencing, they also tested the effect of mutants of additional splicing factors on the silencing of marker genes. Their results indicate that many but not all splicing factors are involved in the centromere silencing. Expression analysis indicates that these splicing mutants with defective silencing have increased *dg* and *dh* transcripts and reduced centromeric siRNA accumulation, which suggests that splicing factors are involved in the siRNA accumulation [49]. One explanation for these observations is that the defective silencing in splicing factor mutants might result from the impaired splicing of mRNA encoding RNAi components. However, their data showed that the splicing of known RNAi components were not affected in the splicing mutants compared to the wild-type, suggesting that the silencing defects in the splicing mutants could not be attributed to the inefficient splicing of the RNAi components [49]. To examine the mechanisms of function of splicing factors involved in the siRNA accumulation, they analyzed immuoprecipitates of FLAG-tagged Cid12, a component of RDRC complex, and found that many splicing factors including those splicing factors involved in centromere silencing were associated with Cid12-FLAG. Therefore, they propose that spliceosomal complexes provide a platform that facilitates RDRC processing of centromeric transcripts for downstream siRNA generation, and thereby promote effective centromere silencing [49].

In a similar study, Chinen *et al.* found that the *prp13-1* mutant with a single nucleotide change on U4 snRNA is defective in centromere silencing, suggesting that splicing factors are involved in the centromere silencing [50]. Their work also confirmed that the processing of the centromeric transcripts into siRNAs were impaired in some splicing factors. Furthermore, they found that the centromeric *dg* and *dh* regions contain intron-like sequences and the spliced form of the *dg* transcript was detected, which suggests that the splicing factors are recruited to the repeat regions through the recognition of the intron sequences [50]. Nevertheless, although the *dg* noncoding RNA can recruit the splicing factors, the siRNAs are generated from both exon and intron regions of *dg* RNA [60], suggesting that the splicing activity of splicing factors may be dispensable for the amplification of centromeric siRNAs.

It is still not clear how splicing factors are recruited. As suggested above, both RDRC and the intron-like sequence of *dg* and/or *dh* RNA may play roles in recruiting the splicing factors to the centromeric regions. It is well known that splicing can occur co-transcriptionally [61,62,63], and the recruitment of splicing factors to the transcription sites can be dependent on Pol II [64]. The *in vivo* interaction of splicing factors with Pol II has also been documented [65]. Therefore, it is also possible that splicing factors are recruited to the centromeric regions via the association with Pol II. Although the spliceosomal complexs are required for the siRNA generation, exactly how splicing factors regulate siRNA accumulation through the association with RDRC is still not clear. Since there are reports showing that splicing factors can regulate Pol II transcripts [66,67], further work is also required to examine whether splicing factors regulate siRNA generation through the influence on Pol II transcription of centromeric transcripts in addition to the proposed model that spliceosomal complexes provide a platform for siRNA generation [49].

## 3. RNA Splicing Factors and RNA-Directed DNA Methylation (RdDM) in Plants

The RdDM pathway in plants parallels the pathway of RNAi-directed silencing process in *S. pombe*. By using *FWA* transgene silencing as a reporter system in *Arabidopsis thaliana*, Ausin *et al.* screened for T-DNA insertion mutants that are defective in the *de novo* methylation of the transgene and identified a line with T-DNA insertion in the *SR45* gene [68]. The *sr45* mutant has decreased DNA methylation in transgenic *FWA* and the associated late flowering phenotype. *SR45* encodes an ARGININE/SERINE-RICH 45 protein belonging to a conserved family of structurally and functionally related, essential pre-mRNA splicing factors. These results reveal that, similarly to that in *S. pombe*, splicing factors are also involved in the RdDM pathway in *Arabidopsis thaliana*. The siRNA accumulation of both type I (dependent on both Pol IV and Pol V) and type II (only dependent on Pol IV) is decreased in *sr45* mutant plants [68]. Furthermore, the level of AGO4 protein that is destabilized in mutants upstream of siRNA biogenesis [40] is also reduced in the *sr45* mutant. These data suggest that SR45 acts at an early step in the RdDM pathway in siRNA generation [68]. Although the indirect effect of SR45 on the expression of genes encoding RdDM components via its RNA splicing function is not excluded in this study, we speculate that SR45 may be directly involved in the RdDM pathway because other splicing factors have been demonstrated to regulate RdDM pathway directly as described below.

How SR45 is recruited and how it regulates siRNA accumulation are still not clear. In *Arabidopsis thaliana*, RDR2 isolated from a pol IV mutant lacks polymerase activity *in vitro*, suggesting that RDR2 is coupled with Pol IV for dsRNA synthesis [69]. It remains to be determined whether like in *S. pombe*, the Pol IV-RDR2 complex and/or Pol IV transcripts participate in the recruitment of SR45. The SR45 regulation of siRNA accumulation might be through regulation of Pol IV transcripts or affecting the processing of Pol IV transcripts into siRNAs.

Recently, Zhang *et al.* carried out a forward genetic screen to identify genes involved in RdDM pathway by using the *RD29A* promoter-driven luciferase (*RD29A-LUC*) reporter system [70]. From this screen, they identified a zinc-finger and OCRE domain-containing protein, ZOP1, required for RdDM. ZOP1 is also a splicing factor based on the evidence that ZOP1 is associated with several typical components of the splicing machinery and that the *zop1* mutant has increased intron-retention events compared to the wild-type [70]. Although many genes have intron retentions in the *zop1* mutant, genes encoding the RdDM components are not in the list. Furthermore, the expression of genes encoding RdDM components is also not affected by the *zop1* mutation. These suggest that the ZOP1 involvement in the RdDM pathway is quite direct, although we cannot exclude the possibility that other unidentified genes involved in RdDM were influenced in their expression by the *zop1* mutation. Similar to that in the *sr45* mutant, Pol IV-dependent siRNA accumulation was reduced in the *zop1* mutant [70]. Additionally, immunofluorescence assay indicated that ZOP1 is present at condensed nucleolus-adjacent foci including the Cajal body as well as dispersed nucleoplasmic speckles, and that ZOP1 partially colocalizes with NRPE1 and DRM2 in the nucleolus-adjacent foci, but not with NRPD1. The Cajal body is the snRNP assembly center [71] and is also required for the assembly of the AGO4 effector complex [72]. Therefore, the authors suggested that ZOP1 regulates RdDM, probably through influencing the assembly of the AGO4 effector complex, or that ZOP1 is associated with NRPE1 and DRM2 of the RdDM pathway.

Interestingly, coimmunoprecipitation assays indicated that ZOP1 interacts with Pol II *in vivo* [70]. Pol II can also be involved in RdDM through its generation of non-coding transcripts that recruit the AGO4-containing effector complex [28,73]. This suggests that ZOP1 functions in the RdDM through the regulation of Pol II. However, it is also possible that the association of ZOP1 with Pol II only reflects a function of ZOP1 in pre-mRNA splicing, but not in RdDM. In addition to ZOP1, the study also identified several other splicing factors affecting siRNA accumulation and DNA methylation [70]. Nevertheless, it remains to be demonstrated whether these splicing factors function in RdDM through a similar mechanism as ZOP1 does.

Using the same screening system, the same research group also identified a PRP6-like splicing factor, STA1, required for the RdDM [74]. STA1 is a U5 snRNP-associated protein and has been demonstrated to be involved in pre-mRNA splicing *in planta* [75]. Northern blot and small RNA deep sequencing analyses indicated that STA1 predominantly influences the accumulation of siRNAs that depend on both Pol IV and Pol V. Furthermore, Pol V-dependent transcripts are partially reduced in the *sta1* mutant [74]. These results suggest that STA1 regulates siRNA accumulation probably by influencing Pol V transcripts. Immunolocalization assays indicated that STA1 is almost exclusively present in the Cajal body and the subnuclear localization largely overlaps with that of AGO4. In addition, STA1 also partially colocalizes with NRPE1 [74]. Therefore, STA1 might act at a late step in the RdDM pathway to facilitate the generation of Pol V transcripts and thereby feedback regulates siRNA biogenesis.

In a similar study, Huang *et al.* identified a U4/U6 snRNP-associated protein, RDM16, which is also required for RdDM [76]. RNA-seq analysis confirmed that RDM16 is involved in pre-mRNA splicing *in planta*. The RNA-seq and mRNA expression analysis also revealed that the pre-mRNA splicing of known RdDM genes is not affected in the *rdm16* mutant, suggesting that RDM16 might be directly involved in RdDM. Nevertheless, unlike the splicing factors described above, mutation of *RDM16* does not affect siRNA accumulation. Chromatin immunoprecipitation (ChIP) assays revealed that RDM16 is enriched at Pol V target loci, suggesting that RDM16 might regulate the expression of Pol V transcripts. Consistently, the Pol V transcripts are reduced in the *rdm16* mutant [76]. The decrease in Pol V transcript levels but not siRNA abundance in the *rdm16* mutant suggests that the reduced levels of Pol V transcripts in *rdm16* may not be strong enough to cause a decrease in the siRNA accumulation. The association of RDM16 with Pol V target loci suggests that RDM16 might be recruited by Pol V and/or the nascent Pol V transcripts. However, it remains to be demonstrated how RDM16 regulates Pol V transcripts. RDM16 may regulate the transcription of Pol V target loci, or the processing or the stability of Pol V transcripts.

**Figure 1:**
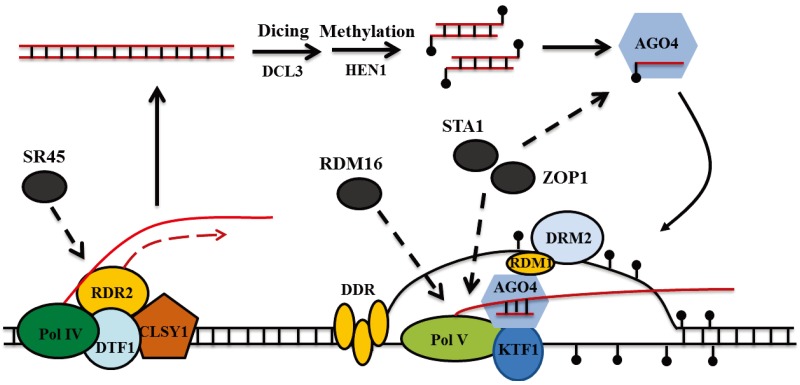
A model for the involvement of splicing factors in different steps of the RNA-directed DNA methylation (RdDM) pathway. With the assistance of CLSY1, Pol IV is recruited to transcribe transposons and repeat loci through an interaction with DTF1/SHH1, which recognizes unmethylated K4 and methylated K9 modifications of histone H3. Coupling of Pol IV and RNA-DEPENDENT RNA POLYMERASE2 (RDR2) is required for copying Pol IV-generated transcripts into dsRNAs by RDR2. The splicing factor SR45 might be recruited by the Pol IV-RDR2 complex and facilitates the siRNA generation. The dsRNAs were diced into 24-nt siRNAs by DICER-LIKE 3 (DCL3), followed by HEN1 methylating the siRNAs at their 3'ends. One strand of the siRNAs is loaded into AGO4 and the siRNA‑bound AGO4 is recruited by Pol V transcript through base-pairing between the siRNA and nascent transcript. The interaction of AGO4 with KTF1 and Pol V is required for the stable association of AGO4 with the Pol V transcripts. DDR complex, which is consisted of DRD1, DMS3 and RDM1 proteins, facilitates Pol V transcription. The interaction of RDM1 with AGO4 and DRM2 may be involved in recruiting DRM2 to Pol V-target loci for catalyzing DNA methylation. The splicing factors STA1 and ZOP1 might be recruited by AGO4-containing effector complex, Pol V or Pol V transcripts and act at a late step in RdDM, while the splicing factor RDM16 might be simply recruited by Pol V or Pol V transcripts to function in RdDM.

The above studies led us to propose that different splicing factors are recruited at different steps of the RdDM pathway (Figure 1). SR45 might be recruited by the Pol IV-RDR2 complex and act early in siRNA biogenesis, whereas ZOP1 might act after siRNA generation. How ZOP1 is recruited remains unclear. Besides the possibility that Pol II might be involved in recruiting ZOP1 for the regulation of RdDM, AGO4-containing effector complex, Pol V or DRM2 may also play roles in the recruitment of ZOP1. STA1 regulates siRNA accumulation as well as Pol V transcripts and it colocalizes with both AGO4 and NRPE1; therefore, STA1 seems to also act at a late step in RdDM and might be recruited by AGO4-containing effector complex, Pol V or Pol V transcripts. RDM16 is enriched at Pol V target loci and regulates Pol V transcripts, suggesting that RDM16 also functions at a late step in the RdDM and that Pol V or Pol V transcripts might be involved in the recruitment of RDM16.

## 4. Conclusion

The involvement of splicing factors in transcriptional silencing and epigenetic regulation seems to be a conserved mechanism in eukaryotic cells. In *S. Pombe*, splicing factors interact with RDRC to facilitate siRNA generation and thereby affect RNAi-directed heterochromatin formation. Nonetheless, the exact mechanisms of function of the splicing factors in the siRNA generation remain to be elucidated. Although the RdDM pathway in plants parallels the RNAi-directed silencing pathway in *S. pombe*, plants have evolved two specialized RNA polymerases, Pol II-derived Pol IV and Pol V, to participate in the RdDM pathway. The increased complexity in the RdDM might lead to the recruitment of splicing factors to different steps of the RdDM to regulate DNA methylation in plants. In the future, it remains to be demonstrated how different splicing factors are recruited to the different steps of the RdDM pathway, and the exact mechanisms of function of the various splicing factors in the RdDM pathway.
